# Distinguishing Local Demagnetization Contribution to the Magnetization Process in Multisegmented Nanowires

**DOI:** 10.3390/nano12121968

**Published:** 2022-06-08

**Authors:** Jorge Marqués-Marchán, Jose Angel Fernandez-Roldan, Cristina Bran, Robert Puttock, Craig Barton, Julián A. Moreno, Jürgen Kosel, Manuel Vazquez, Olga Kazakova, Oksana Chubykalo-Fesenko, Agustina Asenjo

**Affiliations:** 1 Instituto de Ciencia de Materiales de Madrid, CSIC, 28049 Madrid, Spain; jorge.marques@csic.es (J.M.-M.); cristina.bran@icmm.csic.es (C.B.); mvazquez@icmm.csic.es (M.V.); oksana@icmm.csic.es (O.C.-F.); 2 Helmholtz-Zentrum Dresden-Rossendorf e.V., Institute of Ion Beam Physics and Materials Research, 01328 Dresden, Germany; j.fernandez-roldan@hzdr.de; 3 National Physical Laboratory, Hampton Road, Teddington TW11 0LW, UK; robb.puttock@npl.co.uk (R.P.); craig.barton@npl.co.uk (C.B.); olga.kazakova@npl.co.uk (O.K.); 4 Department of Physics, Royal Holloway University of London, Egham TW20 0EX, UK; 5 Physical Science and Engineering Division, King Abdullah University of Science and Technology, Thuwal 23955-6900, Saudi Arabia; julian.moreno@kaust.edu.sa; 6 Computer Electrical and Mathematical Science and Engineering Division, King Abdullah University of Science and Technology, Thuwal 23955-6900, Saudi Arabia; jurgen.kosel@kaust.edu.sa; 7 Silicon Austria Labs, Sensor Systems Division, A-9524 Villach, Austria

**Keywords:** magnetic nanowires, magnetization reversal processes, magnetoresistance, magnetic force microscopy

## Abstract

Cylindrical magnetic nanowires are promising materials that have the potential to be used in a wide range of applications. The versatility of these nanostructures is based on the tunability of their magnetic properties, which is achieved by appropriately selecting their composition and morphology. In addition, stochastic behavior has attracted attention in the development of neuromorphic devices relying on probabilistic magnetization switching. Here, we present a study of the magnetization reversal process in multisegmented CoNi/Cu nanowires. Nonstandard 2D magnetic maps, recorded under an in-plane magnetic field, produce datasets that correlate with magnetoresistance measurements and micromagnetic simulations. From this process, the contribution of the individual segments to the demagnetization process can be distinguished. The results show that the magnetization reversal in these nanowires does not occur through a single Barkhausen jump, but rather by multistep switching, as individual CoNi segments in the NW undergo a magnetization reversal. The existence of vortex states is confirmed by their footprint in the magnetoresistance and 2D MFM maps. In addition, the stochasticity of the magnetization reversal is analysed. On the one hand, we observe different switching fields among the segments due to a slight variation in geometrical parameters or magnetic anisotropy. On the other hand, the stochasticity is observed in a series of repetitions of the magnetization reversal processes for the same NW under the same conditions.

## 1. Introduction

The excellent response of cylindrical magnetic nanowires (NWs) to external stimuli (magnetic, electrical, or mechanical), coupled with their interesting static and dynamic magnetic properties, arises from their high aspect ratio and curved geometry [1]. As such, cylindrical NWs are promising three-dimensional (3D) building blocks for a range of diverse fields [2], including biomedical applications [3,4], spin-based nanoelectronics [5,6], and neuromorphic devices [7,8]. An in-depth understanding of the magnetic states in these nanostructures is thus essential for the implementation of cylindrical nanowires in new 3D technologies. Recently, engineering cylindrical magnetic nanowires leads to unique magnetic configurations and magnetization reversal processes. Particularly appealing are multisegmented nanowires where the proper design of the geometry of the segments and their compositions present many opportunities in magnetic domain configuration and spin dynamics [9,10]. The magnetic properties of these NWs as well as the magnetization reversal process [11,12,13] are tunable through (i) their magnetic material properties (such as magnetocrystalline anisotropy), (ii) the length/diameter ratio of the nanostructure, and (iii) the multilayered induced properties. Moreover, the magnetization reversal can by controlled by alternating the anisotropy or the geometry along the nanowire length [14,15].

Controlling magnetization reversal in each segment creates the opportunity to employ multisegmented nanowires for nanobarcoding [16], shift registers, and magnetic storage applications such as racetrack memories [17]. In this scenario, understanding the specific mechanism that triggers the magnetization reversal process of complex nanostructures is crucial for the development of applications that require precise control of magnetization switching. With this aim, several techniques offer access to local magnetization and its dynamics [18], such as magnetic force microscopy (MFM) [19], magneto-optic Kerr effect (MOKE) [20], Lorentz microscopy [21], holography [22], X-ray magnetic circular dichroism coupled with photoemission electron microscopy (XMCD-PEEM) [23], or magnetotransport measurements [24], which are used to investigate the spin configuration of individual nanostructures and their dynamics, and are largely supported by micromagnetic modeling.

In this paper, we present ordered arrays of multilayered Co_85_Ni_15_/Cu cylindrical NWs synthesized by electrodeposition into nanoporous templates of alumina [25,26]. This technique constitutes an inexpensive, reproducible, and scalable method that allows for its easy implementation in novel applications. The magnetic configuration of these NWs with alternated Co_85_Ni_15_ and Cu segments is explored under the application of an external magnetic field by magnetoresistance (MR) measurements, advanced MFM imaging, and micromagnetic modeling. A multistep magnetization reversal process is observed in these NWs. In addition, the stochasticity of the magnetization process is analyzed.

## 2. Materials and Methods

*Nanowires fabrication:* Co_85_Ni_15_/Cu NWs were grown by pulsed electrodeposition inside the nanopores of anodic aluminum oxide (AAO) membranes [27,28]. The membranes were previously synthesized by hard anodization on high-purity Al foils using oxalic acid (0.3M) as an electrolytic bath with a constant temperature constant of 0 °C [9].

**Figure 1:**
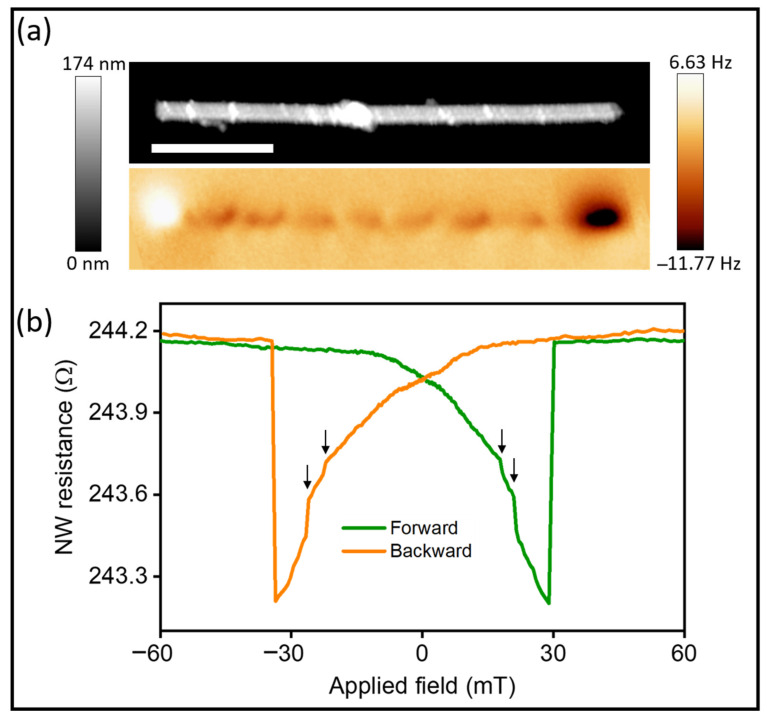
(**a**) AFM and (**b**) MFM image of an individual Co_85_Ni_15_/Cu nanowire. Scale bar = 2 μm. (**b**) Magnetoresistance curves of a nanowire that is under a current of 0.5 mA. Forward direction corresponds to the field applied from −60 to +60 mT, and backward from +60 to −60 mT). Arrows indicate positions of some jumps in magnetization reversal.

The magnetic nanowires were deposited inside alumina pores using an electrolyte: 0.178 M CoSO_4_∙7H_2_O + 0.01 M CuSO_4_∙5H_2_O + 0.095 M NiSO_4_∙6H_2_O + 0.16 M H_3_BO_3_ + 0.06 M C_6_H_8_O_6_. The pH value was kept constant at about 3.0. Different potentials were used during nanowire growth: −1.2 V for 60 s for the NiCo layer and −0.6 V for 30 s for the Cu layer, both versus a Ag/AgCl reference electrode. The resulting multisegmented nanowires with a length of between 7 and 9 µm and diameter of about 100 nm were formed by 700 nm long CoNi segments separated by 30 nm Cu layers (see Figure 1).

For MR and MFM measurements, the AAO membrane was removed, and NWs were dissolved and deposited onto a Si/SiO_2_ substrate by spin coating.

*Magnetic Force Microscopy:* Atomic force microscopy (AFM) and MFM images were recorded using a Nanotec scanning probe microscopy system controlled by WSxM software [29]. The probe used was a Nanosensors PPP-MFMR. The measurements were carried out in amplitude modulation mode, with the phase-lock-loop (PLL) feedback on, thus recording the magnetic signal as the oscillation frequency shifted with respect to the free oscillation frequency. Variable field MFM (VF-MFM) was used to collect nonstandard 2D MFM maps [30].

*Magnetoresistance measurements:* Individual nanowires were electrically contacted in order to perform MR measurements. An electrical contact of Cr (10 nm)/Au (180 nm) was patterned on each end of the nanowire placed on a Si/SiO_2_ substrate. The distance between the two contacts was ~2.5 μm. Additional information about the preparation and fabrication of the electrodes is available in Ref. [31]. The NW was placed between two coils, parallel to the direction of the applied magnetic field. We set an electric DC current of 0.5 mA flowing through the nanowire (i.e., current density of $6.4\times 10^{10}$ A/m^2^). An external field was applied in both directions to the NW, where the field was swept between ±138 mT, in increments of 1 mT, recording the resistance for each field value.

*Micromagnetic modeling:* A micromagnetic model of a representative part of the experimental NWs was created using mumax3 [32]. Three Co_85_Ni_15_ segments of 98 nm in diameter and lengths of 694, 710, and 700 nm were spaced by nonmagnetic layers (simulating the Cu interspaces) with thicknesses of 34 and 26 nm, aiming to introduce symmetry breaking and to reproduce the slight variations in the real parameters of the NWs. The material parameters used were a saturation polarization of 1.6 T, exchange stiffness of $2.6\times 10^{11}$ J/m, magnetocrystalline uniaxial anisotropy of $3.5\times 10^{5}$ J/m^3^ with the magnetocrystalline easy axis at 68° with respect to the NW axis, which corresponded to an hcp structure [14,20,33]. Additional simulations with different anisotropy easy axis directions (between 65° and 70° with respect to the NW axis) and geometry (symmetric wire with all three CoNi segments of 700 nm and Cu spacers of 30 nm) were also performed (see Supplementary Materials).

## 3. Results and Discussion

### 3.1. Multilayered Nanowires

We characterized the isolated NWs after releasing the NWs from the alumina membrane and depositing them onto a Si/SiO_2_ substrate. Figure 1 shows the topography and the MFM image of an isolated multisegmented nanowire. As deduced from the MFM bright/dark contrast at the NW edges (the well-known dipolar contrast), the magnetization of the NW core pointed in the axial direction due to the high shape anisotropy, which counteracted the transversal magnetocrystalline anisotropy. Moreover, the contrast along the nanowire length was due to the nonuniform magnetization of each CoNi segment separated by a Cu spacer layer. The MFM images revealed complex magnetic configurations in each magnetic segment; in some cases, they were compatible with the existence of a configuration of one vortex or two vortices with different chiralities, as previously observed [14,23].

Additionally, magnetoresistance curves were acquired in nanowires, which we electrically contacted for this purpose. A detailed description of the magnetoresistance setup is explained in the Methods section. Figure 1b shows experimental MR curves obtained from sweeping the field from −60 to +60 mT and vice versa. The resistance depended on the relative orientation of the magnetization with respect to the flow of electrons. A decrease in the resistance was expected as the axial projection of the magnetization vector to the current direction decreased, changing from parallel to antiparallel to the current direction. At least two small jumps were observed in each direction (at different absolute values of the magnetic field in the interval from 17 to 33 mT) prior the main jump in resistance, which indicated an irreversible realignment of the magnetization parallel to the nanowire axis (the resistance increased until saturation was reached). As mentioned in the Methods section, the distance between electric contacts was around 2.5 μm, which corresponded to three full CoNi segments interspaced by Cu layers. The jumps in the resistance suggested that the magnetization reversal process was not simultaneously taking place in every individual CoNi segment, but rather varied from one CoNi segment to other. Thus, these features in the magnetoresistance curves can be associated with the magnetization process of single segments.

### 3.2. Magnetization Reversal Process Imaging by Advanced MFM

**Figure 2:**
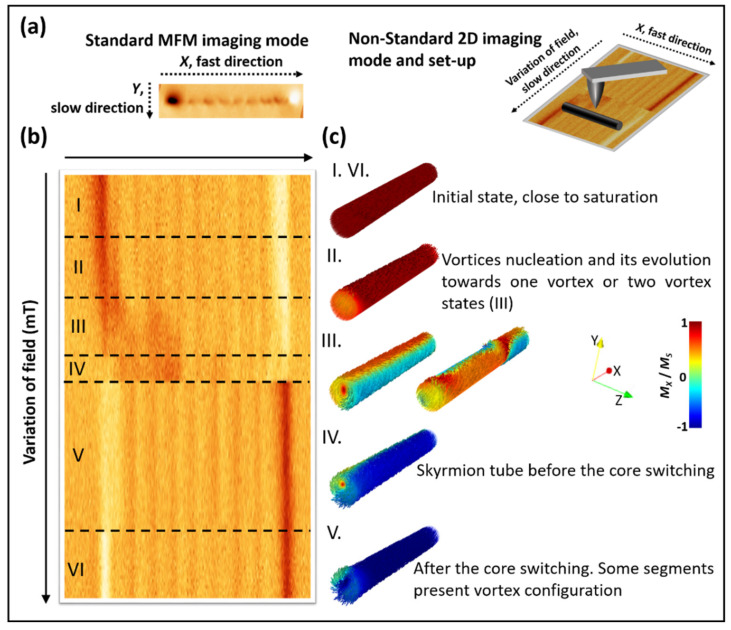
(**a**) Sketch of 2D mode acquisition process; (**b**) 2D MFM map corresponding to a variation of the field between −9 and +37 mT. (**c**) Sketch of the magnetic configuration expected for segments in the regions named as I to VI in (**b**) illustrated using mumax3 data.

MFM is a well-established imaging technique that allows the direct visualization of the magnetization reversal processes of nanoscale systems. The advanced VF-MFM mode used to obtain the 2D magnetic maps is a reliable method to directly image the magnetization reversal processes in elongated nanostructures, and reproduces hysteresis loops of single elements [30]. During the measurements, the same line is repetitively scanned over the long direction of a single nanowire under a varying magnetic field (see sketch in Figure 2a). The field was applied parallel to the *X* scan direction, which was parallel to the nanowire under study. In these 2D mode MFM images, the magnetic field is swept between two saturating field values. In the image obtained, the vertical direction always corresponds to the MFM contrast at same position of the nanowire, whereas the horizontal direction corresponds to the MFM contrast along the nanowires for each value of the applied field.

**Figure 3:**
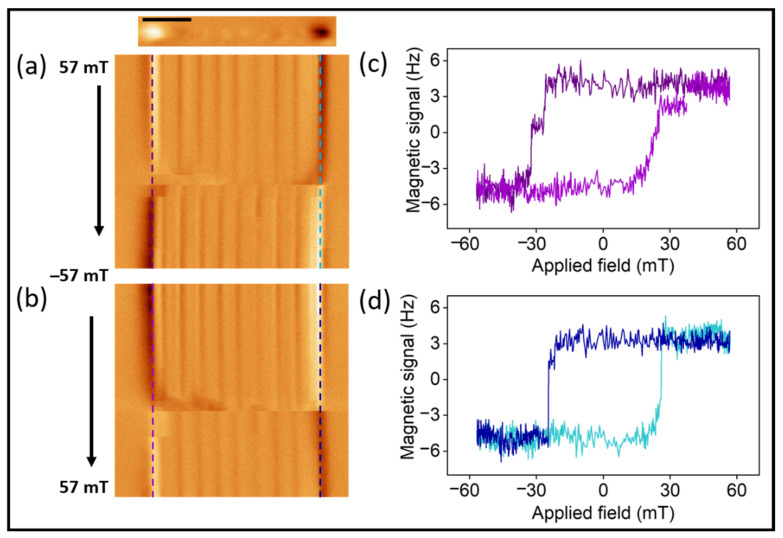
(**a**,**b**) The 2D modes MFM images corresponding to NW measured by MFM in top panel of (**a**). Scale bar is 2 μm. The reconstructed hysteresis curves (measured along the profiles marked in (**a**,**b**)) are presented in (**c**,**d**).

Although the standard MFM contrast contains qualitative information, because it does not obtain direct information about the magnetization vector, the 2D MFM maps provide quantitative information about the switching fields and a breakthrough in high spatial resolution. From the data in Figure 2b, it is possible to determine the nucleation field of the vortex in different CoNi segments, the switching field for the core magnetization, and some information about the evolution of the magnetization in the shell of the NW. Figure 2c illustrates some of these configurations and events that occurred in the different regions (marked as I to VI in Figure 2b) of the 2D MFM experiments. For instance, the close to saturation state (regions I and VI in Figure 2b) of the NW is shown in I in Figure 2c. As the magnetic field decreased, vortex closure configurations nucleated in some segments (region II). In region III, a single or multiple vortex configuration was expected. In region IV, we observed a change in the magnetic state of the first segment of the NW (the one on the left). This change could be attributed to the reversal of the shell magnetization compatible with the skyrmion tube configuration [34]. After the switching of the core all along the NW (between regions IV and V), most of the segments presented a vortex closure configuration at the edges. Two 2D images are shown in Figure 3a,b, each with a different sweep direction of the magnetic field (from + to −57 mT and vice versa). From these images, one can observe that the magnetization reversal process in these nanowires does not show a single Barkhausen jump. Before the main switching field, there were some changes in the MFM signal of different CoNi segments due to the nucleation of a vortex closure or single or multiple vortex states followed by their transformation to skyrmion tubes (see Figure S1 in Supplementary Materials). Significantly, those changes did not simultaneously occur in all the magnetic segments.

By extracting the signal of the stray field along the profiles marked in the 2D MFM maps for both field sweep directions, a nonconventional (local) hysteresis loop was recreated. These curves do not correspond to a hysteresis loop of the whole nanowire, but provide the quantitative value of the switching field. In our particular case, these two hysteresis loops were influenced by the magnetic reversal process of the first segment (the one at the left edge, data shown in Figure 3c) and the last segment (the one at the right edge, data shown in Figure 3d) of the nanowire. In Figure 3c, a clear intermediate step is observed between the complete reversal of the magnetization for each direction of the loop (around −25 and +28 mT), while Figure 3d shows small steps (around −24 and +24 mT). These intermediate steps occurred at different fields (which could be either before or after reaching the coercive field) as can be seen from the loops as well as from the 2D MFM mode measurements (Figure 3a,b), where some inner segments also exhibit a single jump during the magnetization reversal.

**Figure 4:**
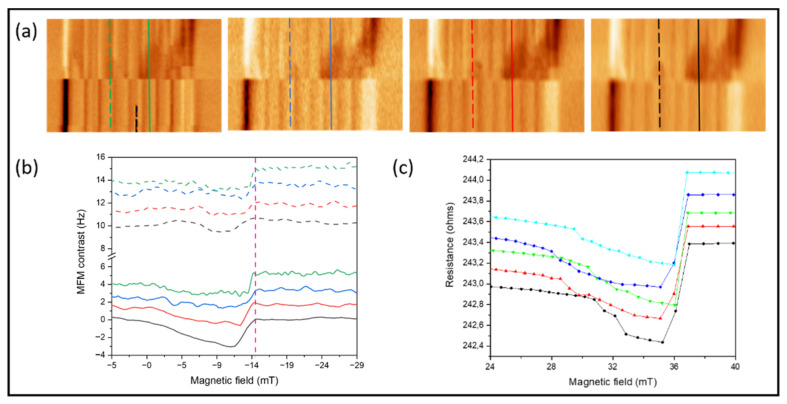
(**a**) A series of 3D MFM images obtained by sweeping the in-plane magnetic field from 8 to −29 mT. (**b**) Profiles obtained along the dashed lines (upper panel) and solid lines (bottom panel). The average switching field is marked by a dashed pink line. (**c**) Successive magnetoresistance curves measured under the same conditions.

Successive 2D MFM measurements were carried out varying the speed and magnetic field amplitude (Supplementary Materials) and extracting local MFM contrast along the same lines in subsequent measurements. Although the switching field value obtained from the 2D MFM images (Figure 3a,b) remained nearly constant for the same segment, small variations in the reversal process of each segment were observed. Figure 4a shows a series of four 2D MFM images corresponding to the magnetization reversal of the same nanowire under the same conditions (same sweeping fields and initial magnetic state). The main features were observed in all the MFM images recorded by sweeping the in-plane magnetic fields between 8 and −29 mT. We concluded that although the reversal process in each CoNi segment (and thus in the whole NW) was almost reproducible, there were small variations in the nucleation fields. The data in Figure 4b obtained from the dashed (upper series) and straight (bottom series) lines marked in Figure 4a demonstrate the existence of slight differences between segments and events. The five curves show similar switching fields, although the nucleation and the magnetization reversal processes vary slightly between events.

This stochastic behavior is in good agreement with the series of magnetoresistance curves (Figure 4c) successively obtained under the same conditions. The presence of several small jumps in the MR curves before the realignment of the magnetization in the NW axis could be explained by the differences in the magnetization reversal processes for the different CoNi segments of the NW, as shown in additional 2D MFM images in the Supplementary Materials. In the Supplementary Materials, we show that slight variations in the length of the segments induced small changes in each reversal field.

### 3.3. Micromagnetic Modelling

**Figure 5:**
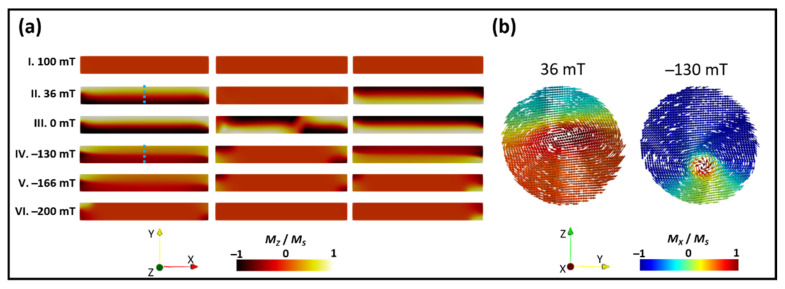
Micromagnetic simulations of a tri-segment nanowire following hysteresis cycle with field applied parallel to NW axis. Nanowire consisted of three CoNi segments spaced between Cu layers (**a**) Magnetization in the z direction of a nanowire at different magnetic field values. (**b**) Cross-sections of the segments are marked in blue in (**a**) for fields of 0 and −130 mT.

To better understand the magnetization reversal process, micromagnetic modeling was performed (see the Methods section) by sweeping the magnetic field from positive to negative saturation along the NW axis. The magnetization components in the *x* and *z* directions (*M_X_* and *M_Z_*, respectively) are shown in Figure 5 in order to distinguish the different configurations. In Figure 5a, we present the evolution of the *M_Z_* component while the field was applied parallel to the NW axis. The *M_Z_* component is shown because the MFM signal was mainly sensitive to the perpendicular component of the stray field. In Figure 5b, we show *M_X_* at the cross-section of the central part of one of the segments (see the blue line in Figure 5a) for two different field values showing configurations close to that of the vortex (left image) and skyrmion (right image).

In these images, with the field sweeping from positive to negative values, at 36 mT, we observed that one of the segments remained saturated, while the other two segments presented a single vortex configuration. Although these structures still presented a high net axial magnetization, a strong reduction in the *M_x_* component was observed because the magnetization of the vortex shell was mainly perpendicular to the axial direction (see Figure 5b at 36 mT). The next magnetization step (from II to III) presented the formation of a domain structure of a pair of vortices in the central segment (see image III) with opposite chiralities (rotational sense). Increasing the in-plane magnetic field, the two-vortex configuration was annihilated, and the single vortex states of the outer segments evolved to a skyrmion tube (see image IV). In this configuration, the x component of the shell magnetization became parallel to the in-plane magnetic field. Note that the core magnetization of the whole NW was still pointing to the right as in the initial state (see Figure 5b at −130 mT). The *M_Z_* in the skyrmion tube was smaller than in the vortex state, in agreement with the decrease in the MFM signal observed in the experiment (see Figure S1 in Supplementary Materials) [34]. After applying a more negative value of field, the complete reversal of the segment cores occurred (compare image IV and image V). In image VI, we observed the existence of closure domains (vortex configuration) at the edges of the NW.

These micromagnetic results highlight that the reconstruction of loops through 2D MFM maps consistently evidences a transformation of a topologically nontrivial magnetic states in the segments of the wire, such as the formation and annihilation of vortex structures and their transformations. Moreover, the modeling shows that the complexity of dynamical transformations and magnetization techniques in multilayered nanowires strongly requires the concurrence of diverse advanced techniques for an unambiguous analysis of the reversal process. Here, the coalescence of nonstandard 2D MFM imaging, MR measurements, and micromagnetic modeling demonstrates that the magnetic reversal mechanism is sensitive to the specific properties of the magnetic segments; thus, distinctions in the vortex nucleation and evolution are observed. However, in the simulations, we observed different switching fields for the segment cores, while in most of the experiments, it seems that the cores simultaneously reversed in all the segments. Some exceptions are shown in Figure S1 in the Supplementary Materials. Notice that by repeating the 2D MFM imaging under the same conditions, we observed slight differences in the magnetization reversal process, indicating stochastic behavior.

Differences between the simulations and the experimental results could be attributed to the geometric asymmetry of the experimental nanowires, i.e., nonhomogeneous CoNi lengths and Cu layers thicknesses, as well as small compositional changes in the crystalline structure (thus affecting the anisotropy direction) along the length of the nanowire. Note also that only three segments were modeled; thus, the strength of dipolar coupling responsible for parallel alignment was diminished. To clarify the importance of experimental inhomogeneity, additional simulations were carried out (see Figure S2 in the Supplementary Materials) varying the anisotropy or the geometry. The results demonstrate the strong influence of those parameters on the magnetization process.

## 4. Conclusions

Cylindrical multilayered CoNi/Cu nanowires were grown by electrodeposition in alumina membranes. The magnetoresistance and MFM experiments allowed us to gain access to the initial stages of the local demagnetization processes. Magnetoresistance measurements showed small jumps in the NW resistance, which suggest that the magnetization reversal does not occur in a single step. Small reversible changes are followed by a large irreversible jump.

VF-MFM imaging and micromagnetic modeling were performed to study the magnetization changes under an applied magnetic field parallel to NW axis. The results show intermediate steps in the magnetization reversal, mainly due to the evolution of the magnetic moment of the shell before the magnetization switching of the core of the segments. This is because the magnetization of all the CoNi segments does not simultaneously reverse; instead, several segments undergo switching of their magnetization at different magnetic field values, ascribed to the slight variations in the specific morphology and composition of each segment. Moreover, stochasticity was observed in the nucleation process of vortices and their evolution with the magnetic field, as deduced from the series of magnetic imaging and magnetoresistance measurements performed under the same conditions. Importantly, we observed slight variations in the switching field, not only through the comparison of different segments (owing to the difference in either geometry or composition) but also by comparing successive repetitions (and thus due to the stochastic behavior). The combination of the MFM technique and micromagnetic modeling allowed us to attribute small jumps in MR measurements to different nucleation fields of vortex structures, while a large jump was associated with a NW core reversal. We envisage that the simple fabrication process and stochastic dynamic properties that we demonstrated here make them a feasible scalable source for low power computation in future 3D neuromorphic nanodevices based on nanowires. Particularly, these applications can benefit from diverse reversal modes and tailoring strategies reported in the literature of the last decade and the development characterization techniques in 3D nanomagnetism.

## Data Availability

The data presented in this work are available upon reasonable request to the corresponding author.

## Supplementary Materials

The following supporting information can be downloaded at: https://www.mdpi.com/article/10.3390/nano12121968/s1, Figure S1: Nonstandard 2D MFM images of a single NW for an applied magnetic field of (a) ±47 mT and (b) ±28 mT; Figure S2: (a) Sketch of two simulated CoNi/Cu NWs with different segments and Cu layer lengths. (b) Hysteresis loops of NWs with different anisotropies (68° and 65° with respect to NW axis) and geometries as shown in (a). (c) Zoom of hysteresis loops of NWs with anisotropy at 65° and different geometries.
