# Supplementary material for: Distinguishing Local Demagnetization Contribution to the Magnetization Process in Multisegmented Nanowires
Source: Nanomaterials (Basel). 2022 Jun 8;12(12):1968. doi: 10.3390/nano12121968 (PMC9229024; doi:10.3390/nano12121968)
Supplement: Supplementary file 1 [file nanomaterials-12-01968-s001.zip › nanomaterials-1739815-supplementary.pdf]

Supplementary Materials

Distinguishing Local Demagnetization Contribution to the Magnetization Process in Multisegmented Nanowires

Jorge Marqués-Marchán [1], Jose Angel Fernandez-Roldan [2], Cristina Bran [1], Robert Puttock [3,4], Craig Barton [3], Julián A. Moreno [5], Jürgen Kosel [6,7], Manuel Vazquez [1], Olga Kazakova [3], Oksana Chubykalo-Fesenko [1] and Agustina Asenjo [1,*]

1 Instituto de Ciencia de Materiales de Madrid, CSIC, 28049 Madrid, Spain; jorge.marques@csic.es (J.M.-M.); cristina.bran@icmm.csic.es (C.B.); mvazquez@icmm.csic.es (M.V.); oksana@icmm.csic.es (O.C.-F.)
2 Helmholtz-Zentrum Dresden-Rossendorf e.V., Institute of Ion Beam Physics and Materials Research, 01328 Dresden, Germany; j.fernandez-roldan@hzdr.de
3 National Physical Laboratory, Hampton Road, Teddington TW11 0LW, UK; robb.puttock@npl.co.uk (R.P.); craig.barton@npl.co.uk (C.B.); olga.kazakova@npl.co.uk (O.K.)
4 Department of Physics, Royal Holloway University of London, Egham TW20 0EX, UK
5 Physical Science and Engineering Division, King Abdullah University of Science and Technology, Thuwal 23955-6900, Saudi Arabia; julian.moreno@kaust.edu.sa
6 Computer Electrical and Mathematical Science and Engineering Division, King Abdullah University of Science and Technology, Thuwal 23955-6900, Saudi Arabia; jurgen.kosel@kaust.edu.sa
7 Silicon Austria Labs, Sensor Systems Division, A-9524 Villach, Austria
* Correspondence: aasenjo@icmm.csic.es

S1. Magnetization reversal imaging by VF-MFM

In Figure 2a, two non-standard 2D MFM images are shown. Figure S1 exhibits additional series of non-standard 2D MFM images with different magnetic field ranges for the same NW studied in Figure 2 data. 2D images from Figure 2a and S1b were recorded with a scan velocity of 1.6 lines per second, while the ones in Figure S1a were recorded at 2.3 lines per second. From this, we conclude that the magnetization reversal processes do not depend on the scan velocity. From these images, it is observed that the magnetization reversal process presents a stochastic behavior since in each scan CoNi segments present slight changes in the reversal field. The advantage of decreasing the range of the 2D MFM experiments is the increase of the resolution. The images in (b) allow to distinguish further details of the magnetization process as the existence of skyrmion tubes (upper panel in (b)) or two vortex configuration in lower panel in (b).

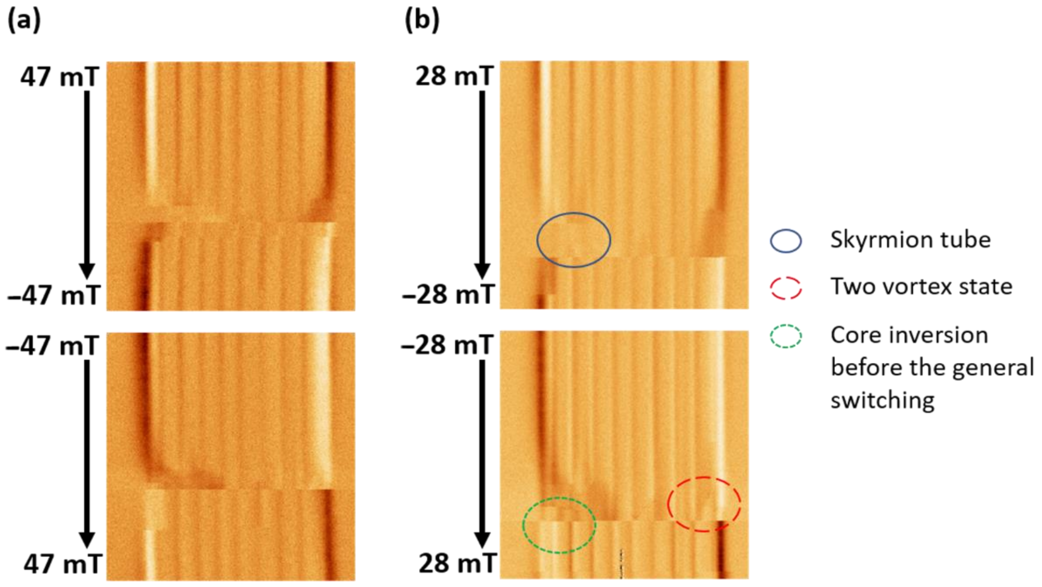

Figure S1. Non-standard 2D MFM images of a single NW for an applied magnetic field of (**a**) ± 47 mT and (**b**) ± 28 mT.

S2. Choice of parameters of micromagnetic modelling

The differences in the reversal magnetization fields for each segment of the NW can be attributed to slight anisotropy differences between CoNi segments and geometrical features. Figure S2 shows three hysteresis loops for NWs with the same (asymmetric) geometry and different anisotropies (68º and 65º with respect to NW axis); and same magnetic anisotropy (65º) but different geometries (Figure S2a). The configuration with asymmetric geometry and anisotropy of 68º corresponds to the same used for micromagnetic modelling in the main text. Experimentally grown NW may not have all CoNi segments nor Cu layers with exactly the same length, but with small variations of some nm. The increase of the anisotropy angle promotes the development of the vortex and skyrmion tube states which are stables in a larger range of fields.

As can be seen in Figure S2c, minimum changes in the simulated NW geometry result in slight alterations of the evolution of the vortex and thus changes in the coercive fields.

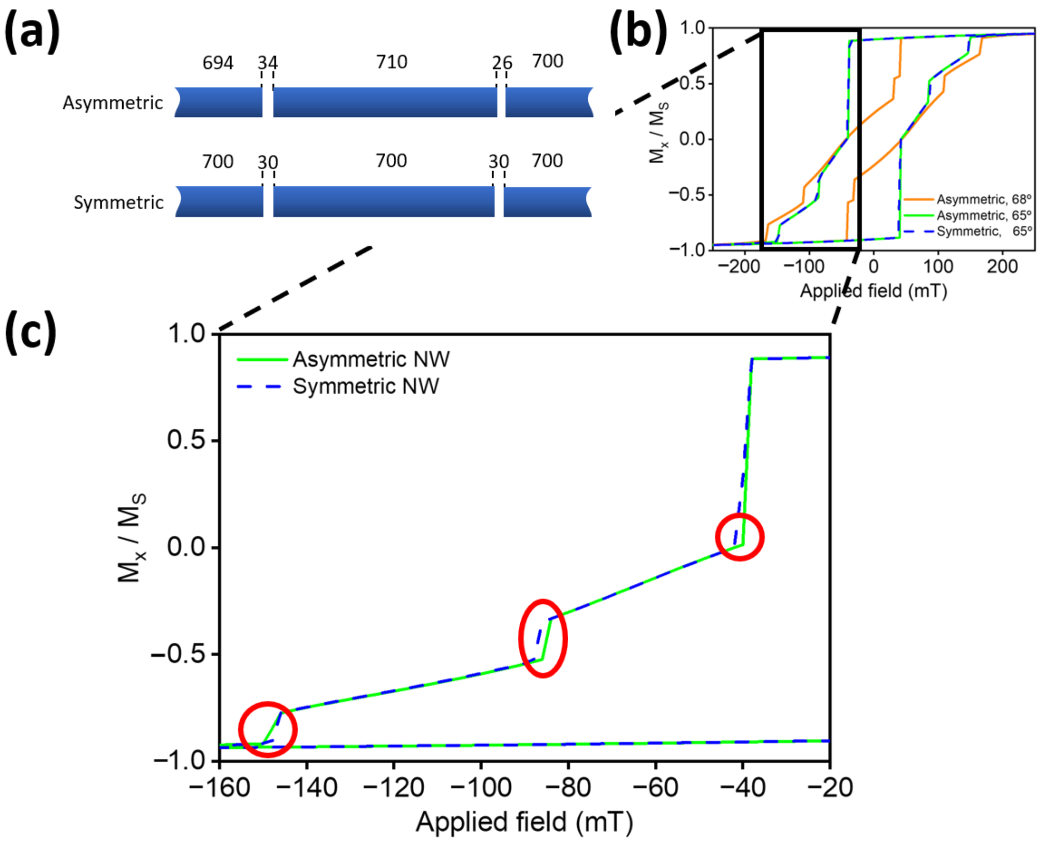

Figure S2. (**a**) Sketch of two simulated CoNi/Cu NWs with different segments and Cu layers lengths. (**b**) Hysteresis loops of NWs with different anisotropies (68° and 65° with respect to NW axis) and geometries as shown in (**a**). (**c**) Zoom of hysteresis loops of NWs with anisotropy at 65° and different geometries.
